# Selection of Appropriate Wound Dressing for Various Wounds

**DOI:** 10.3389/fbioe.2020.00182

**Published:** 2020-03-19

**Authors:** Chenyu Shi, Chenyu Wang, He Liu, Qiuju Li, Ronghang Li, Yan Zhang, Yuzhe Liu, Ying Shao, Jincheng Wang

**Affiliations:** ^1^School of Nursing, Jilin University, Changchun, China; ^2^Orthopaedic Medical Center, The Second Hospital of Jilin University, Changchun, China; ^3^Department of Plastic and Reconstructive Surgery, The First Hospital of Jilin University, Changchun, China

**Keywords:** wound, wound healing, wound dressing, clinical application, physiological mechanism

## Abstract

There are many factors involved in wound healing, and the healing process is not static. The therapeutic effect of modern wound dressings in the clinical management of wounds is documented. However, there are few reports regarding the reasonable selection of dressings for certain types of wounds in the clinic. In this article, we retrospect the history of wound dressing development and the classification of modern wound dressings. In addition, the pros and cons of mainstream modern wound dressings for the healing of different wounds, such as diabetic foot ulcers, pressure ulcers, burns and scalds, and chronic leg ulcers, as well as the physiological mechanisms involved in wound healing are summarized. This article provides a clinical guideline for selecting suitable wound dressings according to the types of wounds.

## Introduction

Physical or thermal damage can cause defects or interruptions in the epidermis of the skin or mucous membranes, forming a wound (Singh et al., 2013). Wounds are classified as acute or chronic wounds. Acute wounds can recover in a short period of time. The size, depth, and degree of injury of the wound are factors that influence the healing process. However, the healing process of chronic wounds is longer and different from that of acute wounds (Schreml et al., 2010). The healing of acute wounds occurs in a normal, orderly and timely manner throughout the entire process. However, the repair of chronic trauma in this fashion is challenging, and it is difficult to restore normal anatomical structure and function (Tarnuzzer and Schultz, 1996; Borda et al., 2016).

There are many factors involved in wound healing (Guo and Dipietro, 2010). The healing process is not static and growth involves four different phases, namely coagulation and hemostasis, inflammatory, proliferation, and remodeling. These phases are not independent but partially overlap on the basis of a sequence by hemostasis, inflammatory, proliferation, and remodeling (Kasuya and Tokura, 2014; Wilhelm et al., 2017). After skin injury, the wound or tissue fracture is filled with blood clots, followed by acute inflammation of the surrounding tissue. The release of inflammatory mediators and infiltration of inflammatory cells cause tissue swelling and pain. Proliferative fibroblasts, endothelial cells, and newly formed capillaries interact to form granulation tissue filling the crevices. During the shaping period, the scars are softened without affecting the tensile strength through the action of various enzymes and stress, thereby adapting to physiological functions (Jeffcoate, 2012; Harper et al., 2014; Nuutila et al., 2016; Ascione et al., 2017a,b).

Medical dressings are essential devices in healthcare. According to the types and stages of wounds, dressings can be applied to their surface and promote healing. The therapeutic effects of traditional dry dressings and modern wet dressings in the clinical management of wounds are documented. Although dressings commonly used in clinical practice (gauze, sterilized absorbent cotton, and bandages) are economical, they can only offer physical protection and have limited benefit on wound healing and prevention of infection. Adherence of the dressing to the wound will cause secondary damage when the two are eventually separated. The generation and development of modern dressings are based on the healing theory of the moist environment and have numerous advantages compared with traditional dressings (Skorkowska-Telichowska et al., 2013; Vowden and Vowden, 2014). For example, modern dressings are conducive to the dissolute and abort necrotic tissue and fibrin, as well as play a role in autolysis and debridement. Moreover, they are beneficial in maintaining a relatively constant local temperature and humidity of the wound, providing the wound with conditions similar to those of the body's internal environment (Richetta et al., 2011; Heyer et al., 2013). Furthermore, modern dressings avoid re-injury of new granulation tissue due to scarring and promote cell proliferation, differentiation, and epithelial cell migration. Particularly, they may play a role in avoiding wound contact with external bacteria and effectively prevent cross-infection (Murakami et al., 2010; Horn, 2012). Although various advanced wound dressings have been developed and applied in the clinical setting, there is no relevant study investigating the reasonable selection of dressing for a certain type of wound (Powers et al., 2016).

**Scheme 1:**
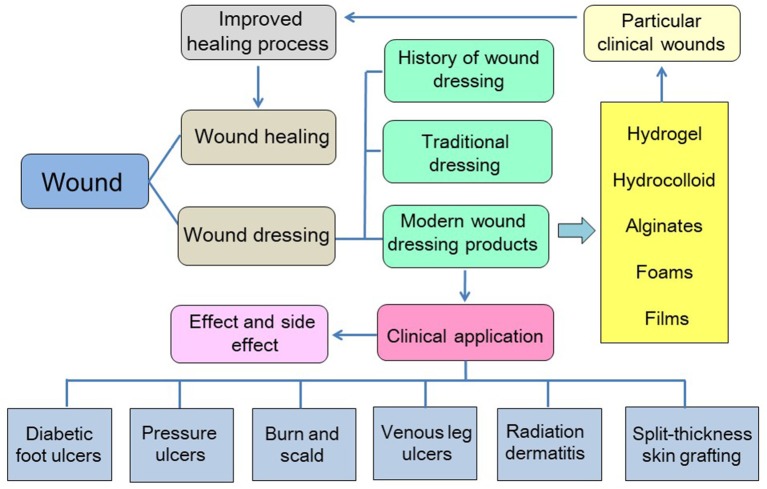
Schematic depiction of the content of this article.

In this review, we summarized the mechanisms of wound healing, traditional and modern wound dressings, and the advantages and disadvantages of both types of dressings. In particular, the clinical application of commercialized modern dressing products in various pathological wounds (diabetic foot ulcers [DFUs], pressure ulcers, burns and scalds, chronic leg ulcers, radiation dermatitis, and skin grafts) is described in detail to provide insight into the care of wounds. The content of this article is shown in Scheme 1.

## Wound Dressings

With the gradual acknowledgment of wound healing theories, the development of wound dressings also evolved considerably. At present, wound dressings are expected to cover the wound and accelerate the healing process (Vowden and Vowden, 2014). Traditional dressings, also termed inert dressings (gauze, cotton pads, and bandages), are the most widely used clinical dressings owing to their low cost and simple manufacturing process (Broughton et al., 2006). However, several shortcomings limit their application, such as difficulty to maintain the wound bed moist and proneness to adhesion to granulation tissue (Moore and Webster, 2013). Modern dressings may be more suitable candidates owing to their properties providing a moist environment for wound healing (Heyer et al., 2013; Moura et al., 2013). Compared with traditional dressings, modern dressings are characterized by better biocompatibility, degradability, and moisture retention. These advantages of modern dressings relieve pain and improve the hypoxic or anaerobic environment (Hopper et al., 2012; Thu et al., 2012; Okuma et al., 2015). The most commonly used modern dressings in clinical practice are hydrogels, hydrocolloid, alginates, foams, and films (Table 1).

**Table 1:** Modern dressings used in clinical practice.

**Variety**	**Description**	**Characteristics**	**Suitable conditions**
Hydrogel	Three-dimensional network of hydrophilic polymers	Moisturizing, removal of necrotic tissue, and monitoring of the wound without removing the dressing	Pressure ulcers, surgical wounds, burns, radiation dermatitis
Hydrocolloid	Hydrogel mixed with synthetic rubber and sticky materials	Excellent exudate absorption properties	Severe exudative wound
Alginate	Consists of polysaccharides derived from brown seaweed	Excellent exudate absorption properties, hemostasis	Infected and non-infected wounds with a large amount of exudate
Foam	Consists of polyurethane or is silicone-based	Semipermeability, thermal insulation, antimicrobial activity	Infected wounds
Film	Consists of adhesive, porous, and thin transparent polyurethane	Autolytic debridement properties, impermeable to liquids and bacteria	Epithelializing wounds and superficial wounds with limited exudate

Hydrogels have a three-dimensional structure composed of hydrophilic substances (Tsang and Bhatia, 2004). They are insoluble in water and subsequently absorb water from 10% to thousands fold their equivalent weight (Goodwin et al., 2016). Owing to their excellent moisturizing ability, hydrogels maintain the wound moist and play a positive role in the cleansing of necrotic tissue. In addition, a wound covered with a dressing can be monitored, as the hydrogels are typically transparent (Hunt, 2003; Scanlon, 2003; Kamoun et al., 2017). Based on these characteristics, hydrogels are primarily used on pressure ulcers, surgical wounds, burns, radiation dermatitis, etc. (Francesko et al., 2017; Shamloo et al., 2018). They are suitable for wounds with minimal-to-moderate exudate. The degradation rate of the hydrogel can also be adjusted, which renders this material appropriate for use as a drug carrier and biologically active substance (Gil et al., 2017). For example, silver nanoparticles (Ag NPs) and ZnO NPs loaded hydrogels can maintain antibacterial activity for a long period of time (Li S. et al., 2018). Recently, a study prepared a multifunctional hydrogel for diabetic wounds. This hydrogel can be used on wounds to collect wound photos via mobile phone and transformed into RGB signals to monitor the pH and glucose levels of diabetic wounds in real time (Zhu et al., 2019). Hydrocolloid and hydrofiber dressings are composed of the same materials in nature. Notably, the latter type is a variant of hydrocolloid dressing appropriate for use as a secondary dressing, which can absorb >25-fold its own weight in fluid while maintaining its integrity (Hobot et al., 2008; Richetta et al., 2011).

Sodium alginate (SA) dressings are fibrous products derived from brown seaweed, which can form a gel after binding to wound exudate (Dumville et al., 2013c; O'Meara and Martyn-St James, 2013). The SA dressings used in the clinic are generally made into sheet fibers, which can be freely cut according to the shape of the wound. SA is also often used to synthesize hydrogels. The SA dressings also possess excellent exudate absorption properties; hence, they can be used in infected and non-infected wounds with a large amount of exudate (Hess, 2000). Owing to the strong absorption property of alginates, their use in the treatment of dry wounds or wounds with minimal exudate should be avoided. Meanwhile, A study developed an alginate hydrogel contained both bioglass and desferrioxamine, which better facilitated diabetic skin wound healing. The results demonstrated that combination use of BG and DFO improved the migration and tube formation of HUVECs as compared with the use of either BG or DFO alone as BG and DFO could synergistically upregulate VEGF expression (Kong et al., 2018).

Foam dressings are semipermeable and either hydrophilic or hydrophobic with a bacterial barrier (Sedlarik, 1994). They are composed of polyurethane or silicone-based, rendering them suitable for handling moderate-to-high volumes of wound exudate (Marks and Ribeiro, 1983). Foam dressings provide thermal insulation and maintain moisture to the wound, and prevent damage to the wound at the time of removal. These dressings may also be used as secondary dressings with hydrogel or alginate dressings, in conjunction with a topical antimicrobial agent for infected wounds (Davies et al., 2017)Moreover, polyaniline/polyurethane foam dressing carried an anti-biofilm lichen metabolite usnic acid indicated an improved antibiofilm activity of conducting polymer (dos Santos et al., 2018).

Film dressings are composed of adhesive, porous, and thin transparent polyurethane. Oxygen, carbon dioxide, and water vapor from the wound pass through the dressing, whereas liquids and bacteria are well-isolated. Furthermore, film dressings possess autolytic debridement properties (Thomas, 1990; Fletcher, 2003), and are suitable for use on epithelializing wounds and superficial wounds with few exudates (Imran et al., 2004). The various types of dressings described above have their own characteristics; thus, the selection of the dressing should be based on the specific conditions of the wound.

## Clinical Applications of Modern Wound Dressing Products

Wound healing involves four different phases, namely coagulation and hemostasis, inflammatory, proliferation, and remodeling (Amini-Nik et al., 2018). Different types of dressings have different characteristics; different pathological types of wounds also have their own characteristics (Table 2). For example, DFUs are prone to infection and cause unsatisfactory wound healing. The prevention of pressure ulcers is focused on the reduction of the shear force and pressure in the hazardous area. Following the formation of the ulcer, it is equally important to prevent further pressure on the ulcer and apply the dressing. Lower extremity chronic ulcers are associated with exudation from wounds due to lower limb edema. Acute wounds, such as burns and scalds, also have their own characteristics. The application of different dressings to different pathological types of wounds in the clinical setting is illustrated in Table 2.

**Table 2:** Overview of various wounds and appropriate clinical dressings.

**Variety**	**Description**	**Characteristics**	**Appropriate dressing**
Diabetic foot ulcer	Caused by neuropathy and lower extremity vascular disease	Lack of supply of oxygen and blood in the wound bed; long-term stagnation in the inflammatory phase	Silver ion foam dressing, hydrofiber dressing, UrgoStart Contact dressing, Mepilex^®^ Lite Dressing, hyaluronic acid, Biatain^®^ Non-adhesive Dressing
Pressure injury	Caused by stress and tissue tolerance	A local injury to the skin or subcutaneous soft tissue occurring at the site of the bone prominence or the compression of the medical device	Foam dressing, hydrocolloids dressing, multi-layered soft silicone foam dressings, polyurethane film, Mepilex^®^ Ag dressing, polyurethane foam dressing
Burn and scald	Tissue damage caused by heat	A large amount of exudate; prone to infection; severe cases can injure subcutaneous and submucosal tissues	Moist occlusive dressing (AQUACEL^®^ Ag), ACTICOAT™ with nano silver
Chronic venous leg ulcer	Caused by high pressure of the blood in the leg veins	Lack of blood supply to the wound; a large amount of necrotic tissue and abnormal exudate on the surface of the ulcer, accompanied by multiple bacterial infections	Alginate dressing, AQUACEL^®^ Ag dressing, Urgotul^®^ Silver dressing, ALLEVYN^®^ Hydrocellular foam dressings, Mepilex^®^ foam dressing
Radiation dermatitis	Local skin lesions caused by radiation	Slow cell proliferation; decreased cytokine activity; decreased collagen content	Film dressing (Airwall), silver-containing hydrofiber, film dressing (3M™ Cavilon^®^ No Sting Barrier Film), Mepilex^®^ Lite dressing
Split-thickness skin grafting	None	Hypertrophic scars; hypopigmentation; hyperpigmentation	Polyurethane foam (ALLEVYN™), calcium alginate (Kaltostat^®^), AQUACEL^®^ Ag (Convatec), Alginate Silver (Coloplast)

### DFUs

In diabetics, the incidence of DFUs is approximately 5–10%. It is one of the most common chronic complications and the cause of lower extremity amputation in patients with diabetes mellitus (Brennan et al., 2017). DFUs as a common type of non-healing or chronic wounds are attracting considerable attention in the medical field (Khanolkar et al., 2008). Currently, the selection of the most appropriate treatment is challenging. During this process, multiple types of dressings are applied to the treatment of DFUs (Saco et al., 2016). One such method is the application of various kinds of modern dressings. Treatment with suitable dressings is an important part of the management of DFUs.

**Figure 1:**
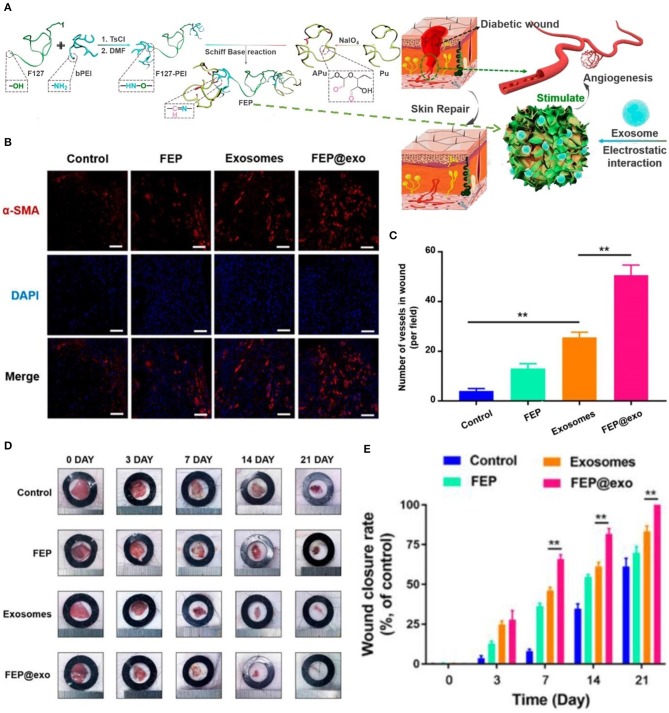
Bioactive dressing promoted angiogenesis in DFU. **(A)** Synthesis and biological function of the fluorinated ethylenepropylene (FEP) hydrogel scaffold containing exosomes. **(B)** Immunofluorescence images of the wound bed stained withα-smooth muscle actin(α-SMA) at day 7. **(C)** The number of new blood vessels at day 7. **(D)** Images of wound healing in mice in different groups. **(E)** Wound closure rate in different groups during wound healing (^**^P < 0.01). Reproduced with permission from Wang et al. (2019).

DFU is defined as foot pain, foot ulcer, and foot gangrene caused by neuropathy and lower extremity vascular disease. The pathogenesis of DFU is very complicated, and its clinical manifestations are heterogeneous (Acosta et al., 2008; Blakytny and Jude, 2009). Therefore, the treatment strategy for DFU is a multi-disciplinary, long-term combination therapy process. Application of dressings is an integral part of long-term treatment options. In the diabetic state, multiple factors cause stagnation in one or more stages of the normal healing process. Microvascular disease results in a reduced supply of oxygen and blood in the wound bed, which delays healing and increases the risk of infection (Rathur and Boulton, 2005; Snyder and Waldman, 2009). Bioactive dressings are a good choice for the repair of diabetic wounds. As shown in Figure 1, researchers have prepared an injectable adhesive thermosensitive multifunctional polysaccharide-based dressing (fluorinated ethylenepropylene) that can continuously release exosomes to promote angiogenesis at the wound site and accelerate the healing process (Khanolkar et al., 2008; Wang et al., 2019). The silver ion foam dressing used in patients with diabetic foot maintains the wound moist. Studies have shown that a better extracellular matrix environment is a vital factor in promoting the migration of keratinocytes and fibroblasts, and synthesis of collagen (Alvarez, 1988; Morton and Phillips, 2012). In addition, silver ions prevent wound infection, thereby avoiding long-term stagnation in the inflammatory phase due to recurrent infections (Barnea et al., 2010).

Several studies have applied modern dressings containing silver ions to the treatment of DFUs. Jude et al. reported the effect of AQUACEL^®^ Hydrofiber^®^ (E. R. Squibb & Sons, L.L.C., Princeton, NJ, USA) dressings containing ionic silver and Algosteril^®^ (Les Laboratoires Brothier, S.A., Nanterre, France) calcium alginate (CA) dressings in patients with diabetes mellitus and non-ischemic Wagner Grade 1 or 2 DFUs. The study found that the clinical effect of ionic silver dressings was better compared with that of CA dressings, especially for the reduction of ulcer depth and healing of infected ulcers. Ionic silver-treated ulcers reduced in depth nearly twice as much as CA-treated ulcers (Jude et al., 2007). Another study used Contreet Foam (Coloplast A/S, Humlebaek, Denmark), a foam dressing containing silver ions to manage patients with diabetic foot. The study showed that Contreet Foam is safe and easy to use, and effectively accelerates the wound healing process (Rayman et al., 2005). A study evaluated the efficacy of hydrofiber dressings and wound healing in DFUs, comparing the safety, final outcome, and patient compliance. Following treatment, hydrofiber dressing showed better healing of the foot ulcer vs. the povidone dressing (Suvarna et al., 2016). Richard et al. studied the effect, tolerance, and acceptability of UrgoStart Contact dressing (Laboratoires Urgo, Chenove, France) in diabetic patients with a neuropathic foot ulcer. The results indicated that the UrgoStart Contact dressing is linked to good tolerance and acceptability, which can effectively promote the healing of neuropathic DFU (Richard et al., 2012).

Zhang et al. compared the efficacy of Mepilex^®^ Lite Dressings (Mölnlycke Health Care, Gothenburg, Sweden) with Vaseline Gauze in the treatment of DFU. The results showed that the study group (Mepilex^®^ Lite) was significantly different from the control group in terms of the mean healing time and wound area. The investigators concluded that the Mepilex^®^ Lite dressing provides a better alternative for the treatment of DFU and warrants further research (Zhang and Xing, 2014). In one study, pure hyaluronic acid was applied to the treatment of DFUs. The results showed that pure hyaluronic acid without other ingredients significantly promotes the healing of DFU without the occurrence of adverse reactions (Lee M. et al., 2016). Lohmann et al. investigated the effect and safety of Biatain^®^ Non-adhesive Dressing (Coloplast A/S, Humlebaek, Denmark) in the treatment of patients with DFU. The results indicated that the average wound area was reduced by more than half in patients treated with the Biatain^®^ dressing (Lohmann et al., 2004). In addition to the common dressings mentioned above, there are other dressings that promote the healing of DFU. A study used sucrose octasulfate dressing treating neuroischemic DFU for 20 weeks, result indicated this dressing significantly improved wound closure without affecting safety (Edmonds et al., 2018). Other study compared bioimplant dressing, a tissue-engineered form of wound dressing containing acellular human amniotic collagen membrane (Life Patch, International Bioimplant Company, Tehran, Iran) with wet dressing in treating DFU. The results show that bio-implantable dressings promote wound healing in DFU better than wet dressings (Edmonds et al., 2018).

DFU is a prevalent and serious global health issue. Wound dressings are regarded as important components of treatment system, with clinicians and patients having many different dressing types to choose from, including hydrogel, foam, hydrocolloid, alginate. The effectiveness of these dressings in DFU has been systematically evaluated, but the conclusions indicated only hydrogels are superior to other types of dressings in healing of DFU (Dumville et al., 2013a,b,c,d). It is worth noting that these systematic reviews included a very small number of studies and were performed several years ago. Decision makers can consider aspects such as the cost of the dressing and the wound management features provided by each type of dressing to determine its use (Wu L. et al., 2015). The effectiveness of these dressings in DFU has been systematically evaluated, but only conclusions are that only hydrogels are superior to other types of dressings in healing of DFU. It is worth noting that these systematic reviews included a very small number of studies and were performed several years ago. Decision makers can consider aspects such as the cost of the dressing and the wound management features provided by each type of dressing to determine its use. It is suggested that more higher quality clinical dressing studies and more comprehensive systematic reviews of the effects of dressings will be conducted in the future.

### Pressure Injury

**Figure 2:**
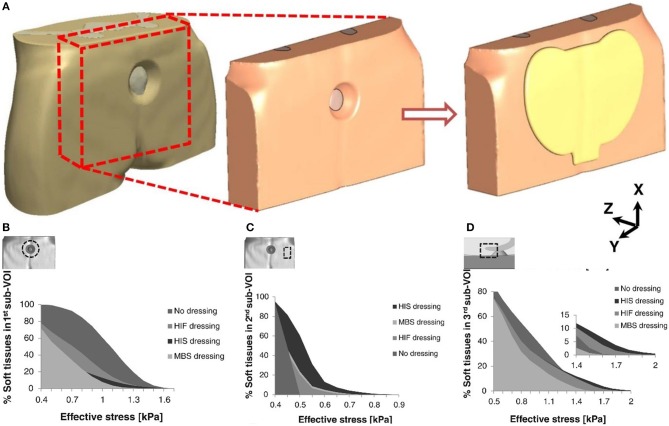
Cumulative volumetric exposures to effective stresses in different parts of the buttocks under combined compression and shear loading. **(A)** Models of the buttock under pressure and coated dressing. **(B)** On the skin surface near the perimeter of the pressure ulcer. **(C)** On the skin surface near the border of the dressing. **(D)** On the skin surface near the tip of the coccyx. Reproduced with permission from Schwartz and Gefen (2019).

Pressure injury is local injury to the skin or subcutaneous soft tissue, manifested as intact skin or an open ulcer, possibly accompanied by pain. It usually occurs at the site of bone prominence or compression of the medical device (Webb, 2017). Stress injuries often occur in patients who are unable to change their position (Pancorbo-Hidalgo et al., 2006; Pieper et al., 2009). The application of dressings is one of the preventive strategies employed in such cases; however, this approach also increases the total cost of treatment. Therefore, it is necessary to determine whether the use of these dressings provides potential benefit to patients (Sebern, 1986). The main factors in the occurrence of injury are stress and tissue tolerance. Stress factors include compressive strength and duration; tissue tolerance is usually affected by the patient's patient's condition and the external microenvironment (Tirgari et al., 2018; Weller et al., 2018). Since the formation of stress injuries can be avoided, prevention is the main task in the clinic. Foam dressings help to reduce the vertical pressure, shear, and friction of the skin, effectively preventing the occurrence of pressure damage (Bolton, 2016; Truong et al., 2016). As shown in Figure 2, researchers have evaluated the effects of the structural and mechanical properties of different dressings to the soft tissue around the wound. These three dressings were Mepilex^®^ Border Sacrum, hypothetical isotropic stiff dressing, and hypothetical isotropic flexible dressing. The anisotropic stiffness feature of the Mepilex^®^ Border Sacrum dressing is essential in wound healing (Schwartz and Gefen, 2019). Studies have shown that excessive skin moisture leads to excessive hydration and damage to the normal barrier function of the skin, hence increasing the risk of ulceration (Demarre et al., 2015). Hydrocolloids or foam dressings for patients with incontinence protect the skin of the appendix from infestation, maintain the skin dry, provide a good microenvironment, and improve tissue tolerance (Williams, 2000). A study assessed the pressure-reducing effect of 10 dressing products, consisting of five types of material (polyurethane foam, hydropolymeric, hydrofiber, hydrocolloid, and low-adherent absorbent). ALLEVYN Non-Adhesive(Smith & Nephew Healthcare, London, UK) exhibited the lowest pressure, while DuoDERM^®^ Extra Thin CGF (ConvaTec Inc., Princeton, NJ, USA)showed the highest pressure (Matsuzaki and Kishi, 2015). Interestingly, a study investigated the modes of action preventing the occurrence of pressure ulcer, such as shear and friction force redistribution, and pressure distribution. The results revealed that the use of Mepilex^®^ and ALLEVYN^®^ dressings reduced frictional forces and shear forces at high-risk areas. In addition, dressings with horizontal fabric structures transferred load over a greater area (Call et al., 2015).

Many clinical studies show that foam dressings can reduce the incidence of pressure ulcers. A randomized controlled trial investigated the role of Mepilex^®^ Border Sacrum and Mepilex^®^ Heel dressings in preventing stress injuries in critically ill patients prior to transfer to the intensive care unit (ICU). The results showed significant differences in the incidence of pressure injuries between the two groups (≤10%). Thus, the study concluded that the application of multi-layered soft silicone foam dressings reduces the incidence in patients prior to transfer to the ICU (Santamaria et al., 2015). A study reported the preventive effect of a five-layer soft silicone border dressing in patients undergoing cardiac surgery in the ICU. The results indicated that there are differences in the occurrence of pressure injury; however, the difference was not significant (Brindle and Wegelin, 2012). Chaiken et al. applied a silicone border foam dressing to the appendix of patients in the ICU to examine whether the dressing reduces sacral pressure injury. The results showed that the incidence of pressure ulcers decreased from 13.6 to 1.8% after application of the dressing, indicating that this type of dressing effectively reduces the incidence of sacral pressure injury in the ICU (Chaiken, 2012). Furthermore, Walsh et al. applied a silicone border foam dressing to the tibia region of patients in the ICU. The results showed that the incidence of hospital-acquired pressure injury in the ICU decreased from 12.5% in 2009 to 7% in 2010, and the number of sacral pressure injury cases decreased from 50 to 13, respectively (Walsh et al., 2012). Nakagami et al. reported a new dressing containing ceramide 2, which can improve the water-holding capacity. The results indicated that the incidence of persistent erythema was significantly lower in the intervention area compared with the control area. The study concluded that the dressing may be applied to patients with thin and dry skin for the prevention of pressure injury (Nakagami et al., 2007). Another study reported the effect of a polyurethane film in preventing postoperative pressure ulcers. The study found that the polyurethane film patch effectively prevented the occurrence of erythema in the sacral area immediately after surgery (Imanishi et al., 2006). A retrospective study investigated the effectiveness of Mepilex^®^ Ag dressings in decreasing post tracheotomy pressure injury. Another retrospective study reported the effectiveness of Mepilex^®^ Ag dressings in preventing stress injuries in children after thoracotomy. Prior to the application of Mepilex^®^ Ag, the incidence of skin rupture during replacement of the first tracheostomy tube was 11.8%. When Mepilex^®^ Ag was applied, there was no occurrence of skin rupture around the stoma. The study concluded that use of Mepilex^®^ Ag reduces the occurrence of postoperative peristomal pressure injury (Kuo et al., 2013). A systematic review evaluated the effectiveness of dressings and topical preparations in preventing pressure ulcers. Nine dressing studies were included in the 18 included studies. It is concluded that silicone dressings can reduce the incidence of pressure ulcers, but the certainty of the evidence is still low and further research is needed to confirm it. At the same time, the role of polyurethane foam dressings and conventional treatments or hydrocolloids in the prevention of pressure ulcers was also compared. Although the results showed no significant difference, the level of evidence in these studies was very low, and more high-quality studies are needed in the future (Moore and Webster, 2018).

Dressings are widely used to treat pressure ulcers and promote healing, and there are many options, including alginates, hydrocolloids, etc. In 2017, a network meta-analysis of dressings and topical medications for pressure ulcers has been performed. This work concluded that there is currently insufficient evidence to determine whether any dressing or topical treatment promotes the healing of pressure ulcers over other methods. However, it is worth noting that many of the trials in this review are small and carry a high risk of bias (Westby et al., 2017). Only one of these studies had a low risk of bias, which compared the effects of local collagen and hydrocolloids on pressure ulcer healing. Although the results showed no significant difference in healing results between collagen and hydrocolloids, the cost of using collagen was more than double that of hydrocolloids (Graumlich et al., 2003).

Although some research results have demonstrated the role of dressings in the prevention and treatment of pressure ulcers. At the same time, the network meta-analysis also revealed generally poor quality of randomized controlled trials of pressure ulcer dressings, which indicates that the trial plan in this field needs to be improved and perfected. Given the uncertainty of the effectiveness of dressing interventions, any investment in future research must maximize its value to decision makers. Any evaluation of future interventions for the healing of compression ulcers should focus on the dressings most widely used by health professionals. In addition, for people with pressure ulcers, faster recovery is as important as whether recovery occurs, so future research should consider the time to recover from pressure ulcers.

### Burns

**Figure 3:**
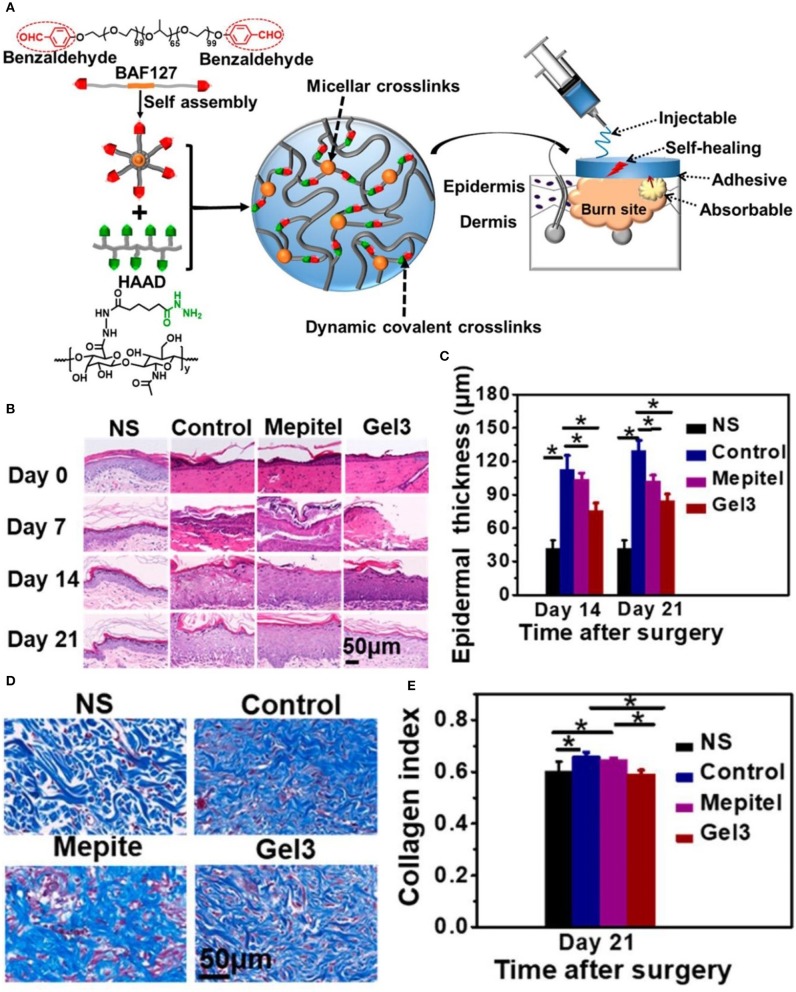
HA-az-F127 hydrogel promotes healing of burn wounds. **(A)** Synthesis and physical characteristics of the HA-az-F127 hydrogel. **(B)** H&E staining at different days after treatment. **(C)** Epidermal thickness in different treatment groups at days 14 and 21. **(D)** Masson's trichrome staining of wounds at day 21. **(E)** Quantification of collagen content in different treatment groups at day 21 (^*^P < 0.05). Reproduced with permission from Li Z. et al. (2018).

Burns, generally caused by heat (i.e., hydrothermal fluids, vapors, hot gases, flames, hot metal liquids or solids) cause tissue damage, mainly on the skin and mucous membranes. Severe cases may also injure subcutaneous and submucosal tissues, such as muscles, bones, joints, and even internal organs (Park, 1978). Acute burns are divided into surface, partial, and full thickness burns (Stavrou et al., 2014). Full-thickness burns involve the entire structure of the skin, and even affect the muscles and bones in severe cases. Despite causing considerable pain and suffering, these types of burns heal easily without surgical intervention. Accurate assessment of the depth of burns is crucial for treatment decision-making. In the presence of infection, superficial and partial thickness wounds can deteriorate into deeper burns. A large amount of exudate causes the patient to lose water and nutrients, and provides the appropriate conditions for bacterial growth. Exudation continues to increase in the inflammatory phase, eventually leading to delayed wound healing. Therefore, most of the dressings (e.g., Ag foam dressings) have the ability of osmotic absorption and prevention of infection. Modern dressings used in the remodeling stage reduce the formation of scars and maximize functional recovery at the wound area. Researchers have prepared a new type of hydrogel, termed HA-az-F127 hydrogel. It is formed by the reaction of a hydrazide-modified hyaluronic acid with a F127 triblock copolymer terminated with a benzaldehyde, as shown in Figure 3. The excellent physical properties of this hydrogel and the action of aspiration drainage promote healing of burn wounds (Li Z. et al., 2018).

Mabrouk et al. compared the effects of two moist wound management methods, AQUACEL^®^ Ag (ConvaTec Inc., Princeton, NJ, USA), a moist occlusive dressing, and MEBO^®^ (Beijing, China), a moist open dressing, in children with facial partial thickness burns. The results showed that the AQUACEL^®^ Ag group had a faster re-epithelialization rate, a lower frequency of dressing change, and less pain, compared with the MEBO^®^ group. The study concluded that the healing rate and long-term outcomes of the moist occlusive wound dressing was better than those of the moist open dressing for the repair of facial partial thickness burns (Mabrouk et al., 2012). A study reported the effectiveness of two commonly used silver dressings, ACTICOAT™ (Smith & Nephew, Hull, UK) and AQUACEL^®^ Ag, in the treatment of partial burns. The results showed that the healing time and bacterial control of the two silver dressings was similar. However, AQUACEL^®^ Ag dressings have advantages over ACTICOAT™ dressings in terms of patient comfort and cost-effectiveness (Verbelen et al., 2014). Bugmann conducted a study to compare the effects of Mepitel^®^ (Mölnlycke Health Care, Gothenburg, Sweden) and silver sulfadiazine for the treatment of pediatric burns. The results indicated that the Mepitel^®^ group achieved a faster healing process (Bugmann et al., 1998). Huang et al. reported the efficacy and safety of the ACTICOAT™ Ag dressing and silver sulfadiazine for the treatment of burn wounds. The study concluded that ACTICOAT™ with nano silver effectively promoted the healing process of residual wounds after burns without the occurrence of adverse effects (Huang et al., 2007).

A study investigated the degree of pain experienced by the patient when using two different dressings: ACTICOAT™ dressing and silver sulfadiazine. The results demonstrated that the use of the ACTICOAT™ dressing for burn wound care is less painful than the use of silver sulfadiazine in patients with partial thickness burns (Varas et al., 2005). A study reported the pain-reducing function of a silver dressing (AQUACEL^®^ Ag) in patients with partial thickness burns. The results indicated that the wound healing time in the AQUACEL^®^ Ag group was significantly shorter compared with that observed in the silver sulfadiazine group. In addition, the patient's pain was also significantly reduced (Muangman et al., 2010). Another study reported the efficacy of an alginate silver dressing, Askina Calgitrol Ag^®^ (B. Braun Hospicare Ltd, Collooney Co. Sligo, Ireland), and 1% silver sulfadiazine in the management of partial-thickness burn wounds. The results indicated that the average pain score and wound healing time in the Askina Calgitrol Ag^®^ group was significantly lower/shorter than those reported in the silver sulfadiazine group (Opasanon et al., 2010).

Similar to DFUs, burns and scalds are generally larger and prone to infection. Therefore, some antibacterial dressings are often used. Silver sulfadiazine is a commonly used wound management method for burns; however, it can easily cause pain in patients. A recent systematic review evaluated the effectiveness of silver-containing foam dressings and traditional SDD dressings in treating partial thickness burns. This work concluded that there is no significant difference in wound healing between silver-containing foam dressing and SSD dressing, but silver-containing foam dressing reduced pain during the early treatment phase and potentially decreased infection rates. Excessive pain may severely affect the patient's mental and physiological state. Therefore, most studies select the severity of pain as one of the outcome variables to compare these two dressings. Rapid healing of wounds and the prevention of hyperplasia of scars in advanced stages of healing are important aspects for patients with burns. Scar hyperplasia in key areas will seriously affect the patient's physiological function and quality of life. Nevertheless, very few studies have focused on this aspect. Future studies including larger sample sizes and follow-up of patients with wound scar hyperplasia are warranted.

### Chronic Venous Leg Ulcers (VLU)

Venous leg ulcers (VLU) are chronic ulcers caused by excessive venous pressure in the lower extremities and abnormal venous blood flow, eventually leading to the formation of an ulcer on the skin of the lower leg (Palfreyman et al., 2007; Chapman, 2017). It is one of the clinical manifestations of chronic venous insufficiency at the most severe stage. The underlying causes of the disease are venous valve incompetence and calf muscle pump insufficiency, leading to venous stasis and hypertension (Gianfaldoni et al., 2017). In this case, the local blood circulation is altered, and the blood supply to the local tissue is insufficient (Serra et al., 2016). Prolonged care leads to high treatment costs. Moreover, the quality of life of patients with chronic VLU is severely affected (Salome et al., 2016).

**Figure 4:**
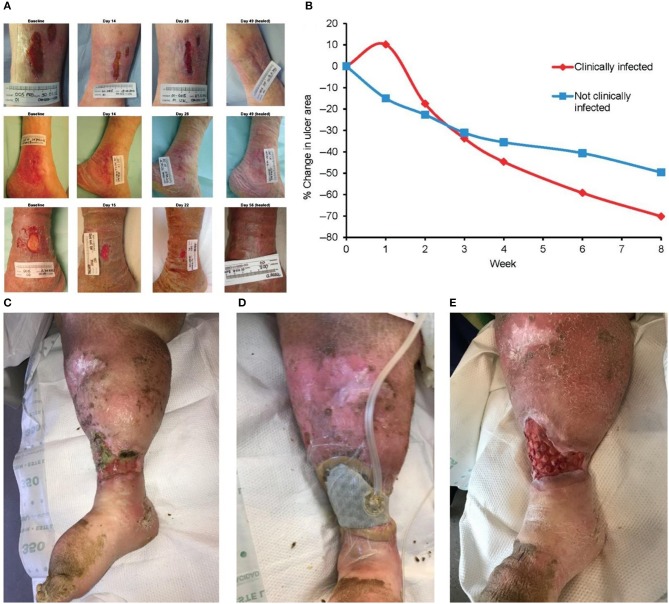
Modern dressings promoting the healing of VLU. **(A)** Ulcer areas in patients with infected (red line) and non-infected (blue line) at different time points. **(B)** Trends in the ulcer area in different patients. **(C)** Initial state of the wound. **(D)** Dressing application of cell foam dressing with through holes (ROCF-CC). **(E)** Dressing replacement. Reproduced with permission from Harding et al. (2016) and McElroy et al. (2018).

The venous regurgitation disorder, insufficiency of the vascular function, weak venous wall, and incomplete systolic muscle pump function are considered to be the main causes of VLU formation (Lozano Sanchez et al., 2014). The inflammatory response of leukocytes and endothelial cells is important in the development of VLU (Raffetto, 2009). Based on the above, skin capillary damage, local microcirculation and tissue absorption disorders, fibrin exudation, accumulation of metabolites, lower extremity edema, and skin nutrition changes, followed by bacterial and other microbial infections, eventually lead to the development of ulcers (Dawkins, 2017). Compression therapy is the main conservative treatment of VLU. The treatment mainly includes bandages, elastic stockings, and inflation and compression devices (Rajendran et al., 2007). Moreover, there is substantial necrotic tissue and abnormal exudate on the surface of the ulcer, often accompanied by multiple bacterial infections. Thus, treatment of the wound surface is also necessary. The Ag foam dressing absorbs a large amount of exudate, and it can be used for the prevention of infection. The dressing can be combined with compression therapy to promote wound healing. The alginate dressing absorbs large amounts of exudate and is also suitable for the treatment of VLU. As shown in Figures 4A,B, silver ion dressing plays a positive role in wound healing (Harding et al., 2016). Of course, debridement is inevitable. A new type of porous mesh foam dressing, cell foam dressing with through holes (ROCF-CC), was introduced into negative pressure wound therapy with instillation and dwell. As shown in Figures 4C–E, this dressing is highly effective on debridement (McElroy et al., 2018).

A study evaluated the effectiveness of knitted viscose and hydrocolloid dressings for venous ulceration. The results indicated that there are no significantly differences in these two dressings (Nelson et al., 2007). Maggio et al. tested the effectiveness and safety of Vulnamin^®^ gel (Errekappa, Milan, Italy) and compressive bandages in patients with lower limb chronic venous ulcers. The results indicated that the use of Vulnamin^®^ together with elastic compressive bandages is safe and more effective than standard dressing (Maggio et al., 2012). Another study compared the wound healing efficacy of AQUACEL^®^ Ag dressing and Urgotul^®^ (Laboratoires Urgo, Chenove, France) Silver dressing for the treatment of venous ulcers at risk of infection. The results showed that both silver dressings were effective in the healing of venous ulcers (Harding et al., 2012). Lammoglia et al. reported the effectiveness and safety of *M. tenuiflora* cortex extract (MTC-2G) in patients with VLU. The results indicated that there was no significant difference between hydrogel containing MTC-2G and hydrogel alone for the treatment of VLU (Lammoglia-Ordiales et al., 2012). A study evaluated LyphoDermTM (XCELLentis, Belgium) gel containing allogeneic epidermal keratinocytes in the treatment of patients with venous ulcers, which are difficult to heal. The results indicated that, in the subgroup with enlarging ulcers, there were significantly more healed ulcers in the LyphoDerm™ group vs. the control group (Harding et al., 2005). Franks et al. compared the effectiveness of ALLEVYN^®^ Hydrocellular and Mepilex^®^, two commonly used foam dressings, in the treatment of chronic VLU. Although the results did not reveal significant differences in the number of patients achieving complete repair of ulcers between the two groups, both dressings reduced pain after treatment (Franks et al., 2007). Another study compared the treatment effect and cost-effectiveness of silver-containing and non-silver low-adherence dressings in the management of VLU. The results indicated that there were no significant differences between the silver-containing dressing group and the control group (Michaels et al., 2009). A study evaluated the effectiveness and safety of Contreet Foam, a dressing with sustained release of silver, in the management of chronic VLU with moderate and high exudation. The results indicated that Contreet Foam combined with silver achieved excellent exudate management in patients with hard-to-heal chronic VLU (Karlsmark et al., 2003).

Unlike the aforementioned types of wounds, VLU in the lower extremities requires treatment of lower extremity edema to promote wound healing. Tissue edema can stress the arteries and affect the blood circulation in the lower extremities, resulting in insufficient blood supply to the wound. The combination of wound dressings and multiple lamination treatments may exert the best therapeutic effect. At the meantime, a network meta-analysis show that silver-containing dressings can increase the likelihood of VLU healing, but because of the small number of related studies and high risk of bias, the most effective treatment is still not determined (Norman et al., 2018). This results of this network meta-analysis focus exclusively on complete healing, did not take other important outcomes into consideration. Therefore, decision makers can appropriately draw on the results of the above studies according to the actual situation of the wound when choosing a dressing. At the same time, more high-quality research is needed in order to obtain more definitive evidence-based evidence in order to provide reliable decision-making basis for decision makers.

### Radiation Dermatitis

Radiation therapy is a common method for the treatment of cancer. It is used to treat cancer that is not suitable for surgery or assist surgery (Terasawa et al., 2009). Radiation-related skin lesions are most common in radioactive local lesions and can be classified as acute radiation-induced skin injury, chronic radiation-induced skin injury, and radiation skin cancer (Kirkwood et al., 2014). Skin side effects of radiation therapy occasionally limit its application (Wickline, 2004; Hird et al., 2008). Severe adverse skin reactions may affect further treatment. At present, the prevention and management of radiation-induced skin injury remains a challenge. Modern wound dressing can be used as a prevention and management method.

Radiation increases the expression of apoptosis-related genes, retards cell proliferation, and decreases cytokine activity and collagen content, resulting in delayed wound healing (Zhang et al., 2012, 2014). A transparent film dressing can be used to protect the skin in the illuminated area. The film dressing using Airwall exhibited a satisfactory prophylactic effect (Arimura et al., 2016).

Radiation dermatitis severity was reduced in patients with breast cancer radiotherapy after prophylactic use of Hydrofilm (Paul Hartmann AG, Heidenheim, Germany) compared with control (Schmeel et al., 2019). A study examined the effect of a film dressing (Airwall) in the management of acute radiation dermatitis induced by proton beam therapy. The results indicated that the Airwall group experienced less severe acute radiation dermatitis compared with the standard management group (Arimura et al., 2016). Perea et al. evaluated the effectiveness of silver-containing Hydrofiber^®^ dressings in minimizing or preventing radiation-induced dermatitis. They suggested that silver-containing Hydrofiber^®^ dressings are effective in reducing radiation dermatitis and arresting its progression, consequently leading to shorter healing time (Whaley et al., 2013). A clinical study investigated the effects of film dressings 3M™ Cavilon^®^ No Sting Barrier Film (3M, Minneapolis, MN, USA), and topical corticosteroids on skin exposed to radiotherapy and compared the effects of the two methods in preventing radiation dermatitis. The results showed that although 3M™ film dressings and corticosteroids were not significantly different vs. control in all respects, 3M™ film dressings may reduce skin itching, while corticosteroids may delay the onset of severe skin inflammation (Shaw et al., 2015). In a single-blind, randomized controlled trial for the prophylactic use of a silicone-based film forming gel dressing (StrataXRT^®^ Stratpharma AG, Basel, Switzerland) in patients with head and neck cancer undergoing radiation therapy, the results show that it is effective for preventing, and delaying the development of grade 2 and 3 skin toxicity (Chan et al., 2019).

For skin already suffering from radiation dermatitis, the use of a suitable dressing can promote healing. Lee et al. studied the effects of a foam dressing combined with recombinant human epidermal growth factor on the treatment of seven patients with head and neck cancer experiencing radiation-induced dermatitis. The wounds of these seven patients with radiation-induced dermatitis healed within 14 days (Lee J. et al., 2016). A study compared the effects of Mepilex^®^ Lite dressing on wound healing and the quality of life in patients with nasopharyngeal carcinoma. The results indicated that the patients in the Mepilex^®^ group had significantly shorter wound healing time and improved sleep quality compared with those in the control group (Zhong et al., 2013).

The onset time of chronic dermatitis usually occurs after radiotherapy for a prolonged period of time (Spalek, 2016). Therefore, the application of modern dressings in radiation dermatitis is mostly focused on acute dermatitis. Although the above studies have concluded that the use of modern dressings and growth factors can improve radiation dermatitis, there is a lack of evidence-based, randomized, controlled trials comparing different types of these dressings. Importantly, future studies should examine skin-specific quality of life and cost-effectiveness. Medical staff should focus on the prevention of radiation dermatitis. They can comprehensively evaluate the skin in the radiotherapy area prior to radiotherapy and use film dressings or liquid dressings to protect the skin.

### Split-Thickness Skin Grafting (SSG)

SSG is a common reconstructive technique used to repair orthopedic wounds and burns. However, the repair and regeneration of the donor site is overlooked, causing unnecessary pain to the patient (Shoemaker, 1982; Kirsner et al., 1997; Coruh and Yontar, 2012). In recent years, the application of new dressings is one of the common methods used to promote the repair of the donor site (Malakar and Malakar, 2001). Studies have shown that as many as half of donor sites show signs of infection, and patients often experience pain at these sites. Leakage of blood and fluid is also common. Infections, pain, and leakage are factors that complicate and retard the healing process, as well as cause hypertrophic scars and hypopigmentation or hyperpigmentation. Therefore, appropriate management of the donor site after the collection of SSG is essential. The application of the dressing is a key part of this process. The ideal dressing should assist rapid epithelialization, prevent infection and leakage, and feel comfortable and painless for the patients. It is also adjustable according to different parts, easy to use, and cost-effective.

The skin graft donor site is a type of surgical wound; therefore, it is less likely to be infected than the aforementioned types of wounds. Dressings used in this condition provide a good healing environment to prevent wound infection and reduce the formation of scars. Researchers have combined antimicrobial-impregnated dressing with negative-pressure wound therapy to greatly improve the survival rate of skin grafts (Wu C. C. et al., 2015). Alginate dressings, hydrocolloid dressings, and foam dressings are used in this setting. A study compared the effectiveness of two types of advanced dressings, namely polyurethane foam (ALLEVYN™) and CA (Kaltostat^®^), in the management of the donor site after SSG. The results indicated that, although there were no significant differences in wound healing time, pain intensity, length of stay, and staff and patient satisfaction between the ALLEVYN™ group and Kaltostat^®^ group, the former dressing was more cost effective than the latter (Higgins et al., 2012). A study compared the effectiveness of two silver dressings, AQUACEL^®^ Ag (Convatec) and Alginate Silver (Coloplast), in the management of donor site wounds. The results showed that Alginate Silver exhibited superior performance in terms of pain and re-epithelialization time (Ding et al., 2013). A trial compared the effectiveness of six wound dressings, including semipermeable film, alginate, hydrocolloid, gauze dressing, hydrofiber, and silicon, in the management of donor-site wounds. The results showed that the hydrocolloid group had the fastest epithelialization rate, and the wound infection rate in the gauze group was 2-fold higher than that reported in the other five groups (Brolmann et al., 2013). A study compared the effectiveness of banded dressings and not banded dressings in patients who underwent skin grafting. Studies showed that the use of polyurethane foams and elastic tape was a simpler but effective method of trimming and may be associated with a shorter operating time than conventional fixation methods using bonded pads (Yuki et al., 2017).

SSG, as a reconstructive technique, is used in burn patients with larger wound bed. The goal of donor site management is to achieve a faster healing speed without pain. Treatment of donor site wounds after SSG is an important clinical issue because patients generally report greater pain at the donor site than at the graft receiving site (Voineskos et al., 2009). Acute wound pain has been shown to increase patient stress and subsequently negatively affect quality of life and lead to delayed wound healing (Broadbent et al., 2003). An evidence-based review summarizes the current evidence that wet wound healing dressing products have clear clinical advantages over non-wet dressing products in treating SSG donor site wounds (Brown and Holloway, 2018). However, no clear trend was detected regarding the performance of each dressing type. So far, there has been limited discussion about the influence of secondary dressings as well as methods/techniques of primary dressing use on donor site wounds. Further research is clearly needed in this area. Especially should explore the role of secondary dressing use, and using more than one primary dressing product throughout the donor site wound-healing process should be taken into consideration.

## Prospect

With the increase in the incidence of diabetes and chronic vascular diseases, wound management (especially for certain chronic wounds) has gradually attracted the attention of clinicians. The poor healing of wounds results in pain to patients and causes a heavy medical burden. For example, DFU can cause severe and persistent infections and, in extreme cases, lead to amputation. The use of dressings is a common treatment for the management of wounds. In particular, modern dressings are superior to traditional dressings in preventing infection, accelerating wound healing, and reducing pain in patients. The selection of the most appropriate modern dressing product is a challenge for clinicians. An ideal dressing should have the ability to maintain moisture balance in the wound, promote oxygen exchange, isolate proteases, stimulate growth factors, prevent infection, facilitate autolytic debridement, and promote the production of granulation tissue and re-epithelialization (Moura et al., 2013).

Although these modern dressing products are superior to traditional dressings in some respects, their cost is higher than that of traditional dressings. The use of modern dressings in countries and regions where health insurance systems are not well-established involves a significant cost, especially for those with low- or average-income levels. Therefore, dressing manufacturers improve production efficiency, optimize production processes, and reduce costs to ensure that more patients benefit from the use of these new dressings. At the same time, research on a variety of new materials for wounds has emerged, but few have been applied to the clinic in the end. Therefore, promoting the industrialization of scientific research results and providing patients with more alternative dressings is a problem that needs to be solved. Most of the studies discussed above were conducted in hospitals and the subjects were hospitalized patients. Nevertheless, chronic wounds (e.g., DFUs and PUs) were treated at home or nursing home in most cases. It is suggested that how to promote wound healing in a home and nursing home should be studied in the future. In particular, most studies have only evaluated the effect of a single dressing on the wound, but it may have better results when combined with other treatments, such as light therapy and topical drugs. It is suggested that this research direction can be considered in the future. At the same time, additional multi-center, high-quality, randomized, controlled clinical trials are warranted to prove the advantages of modern dressing products in wound healing. Last, systematic review and meta-analysis of DFU and pressure ulcers is slightly lagging, and it is recommended to include research in recent years for timely updates to provide reliable evidence for decision.

## Conclusion

In summary, the process of wound healing is not static. It requires an appropriate environment at each stage of the healing process, and a reasonable approach to the selection of dressing for certain types of wounds should be clarified for clinical professionals. In the opinion of the author, an ideal dressing is expected to possess the capacity of moisture balance, promote oxygen exchange, isolate proteases, stimulate growth factors, prevent infection, facilitate autolytic debridement, and promote the production of granulation tissue and re-epithelialization. However, currently, there are no dressings that can achieve all these functions. Hence, the specific selection of modern wound dressings for different wounds should be based on the particular conditions, such as the patient's primary disease, the characteristics of the dressing, and especially the physiological mechanisms of wounds. This article summarized the advantages of various wound dressings and their applications in different wounds, aiming to provide a clinical guideline for the selection of suitable wound dressings for effective wound healing.

## Author Contributions

JW and YS conceived and coordinated this project. CS and CW wrote this paper. RL and YZ collected and summarized literatures. QL and YL edited pictures in this paper. HL revised this paper.

### Conflict of Interest

The authors declare that the research was conducted in the absence of any commercial or financial relationships that could be construed as a potential conflict of interest.
